# Practical diagnostic approach to assess myeloid and precursor cell neoplasms on trephine bone marrow biopsies: reflection of middle European reality

**DOI:** 10.1007/s12308-025-00663-5

**Published:** 2025-10-17

**Authors:** Thomas Menter, Alexandar Tzankov

**Affiliations:** Institute of Medical Genetics and Pathology, University Hospital Basel, University of Basel, Schönbeinstrasse 40, Basel, Switzerland

**Keywords:** Trephine bone marrow biopsy, Histopathology, MPN, Systemic mastocytosis, MDS/MPN, MDS, AML, BPDCN, ALL

## Abstract

Trephine bone marrow biopsies (TBMB) are standard specimens for diagnostics and follow-up of myeloid and precursor cell neoplasms, usually as part of a multimodal approach including complete blood count (CBC) tests, cytologic assessment of BM aspirate smears, flow cytometry, cytogenomics, and molecular genetics. Incorporating the results of all the above methods or running all tests within one specialized lab and providing an integrative report is ideally desirable. However, in reality, in many countries, (1) diagnostic (hemato-)pathologists may not have access to information on the results obtained by other technologies except for a CBC, (2) individual procedures may not be available at all, (3) specimens may not be submitted to specialized labs, or (4) particular technologies may fail yielding results (e.g., dry taps or specimens with destroyed nucleic acids), so that the TBMB might remain the only analytic sample. The detailed description of our approach to handling TBMB including classic histopathology, immunohistochemistry, fluorescence in situ hybridization, and sequencing techniques in the setting of myeloid and precursor neoplasms may help the readership to achieve a comprehensive TBMB diagnostic approach reaching more definite and robust conclusions to improve patient care.

## Introduction

Trephine bone marrow (BM) biopsies (TBMB) are an essential part of the multimodal diagnostic procedures to diagnose hematological diseases. Typically, not only a sample for histology is taken, yet materials are also obtained for cytologic evaluation on aspirate smears, for flow cytometry as well as genetic analyses (cytogenetics, mutational analyses).

Incorporating the results obtained by the different disciplines mentioned above or running all tests within one specialized lab and providing an integrative report is ideally desirable. However, in reality, many obstacles are faced: in many countries, the diagnostic (hemato-)pathologists may not have access to information on the results obtained by other technologies except for a complete blood count (CBC); several, especially molecular procedures, may not be available at all; specimens may not be submitted to specialized labs; and—last but not least—some procedures may fail yielding results (e.g., dry taps or specimens with destroyed nucleic acids), so that the TBMB might remain the only analytic sample.

This review addresses practical diagnostic approaches to myeloid neoplasms (MN) and precursor cell neoplasms on TBMB, including in situ [histopathology, special stains, immunohistochemistry (IHC) (for a summary of purposeful IHC panels see Table 1) and in situhybridization (ISH)] and molecular in vitro methods [sequencing, especially high-throughput sequencing (HTS)] applicable to such specimens.

**Table 1:** Proposal for immunohistochemistry panels for various entities in TBMB

Suspect disease/disease category (to which all pre-test information is in favor of)	Recommended panels with explanatory notes
CML	CD34 (to count blasts), CD42b or CD61 (to detect dwarf megakaryocytes), CD71 or E-cadherin (to prove decreased erythropoiesis), Mast cell tryptase (MCT; to exclude SM-AHN)
MPN, other than CML	CD34 (to count blasts and analyze bone marrow microvessels), CD42b or CD61 (to detect and analyze megakaryocytic clusters), CAL2 (if *CALR* gene status is unknown), phospho-STAT5 (megakaryocytic nuclear staining being a surrogate marker for MPN-driver gene mutations), MCT (to exclude SM-AMN)
CNL	CD34 (to count blasts and analyze bone marrow microvessels), CD14 (to exclude increased monocytes), IRF8 (to detect nodules of mPDC that may be suggestive of MDS/MPN)
SM	MCT and CD117 (to detect MC); CD2 (in combination with CD3 to subtract T-cells), CD25 and CD30 (to detect abnormal MC phenotype) CD14 (to exclude increased monocytes in SM-CMML), CD34 (to count blasts and detect dysplastic megakaryocytes in SM-AMN), IRF8 (to detect nodules of mPDC in SM-CMML and MDS/MPN)
MDS/MPN	CD34 (to count blasts), CD14 (to detect increased, i.e. ≥ 15%, usually M1, monocytes in CMML), IRF8 (to detect nodules of mPDC particularly in CMML), MCT (to exclude SM-AMN)
MDS/MPN-N (aCML)	CD34 (to count blasts), CD14 (to exclude increased monocytes), CD15 (to prove increased myelopoiesis)
MDS	CD34 (to count blasts and detect dysplastic megakaryocytes), CD42b or CD61 (to detect dysplastic and peritrabeculary displaced megakaryocytes), CD71 or E-cadherin (to detect dysplastic and peritrabeculary displaced erythropoiesis), p53 (to detect strongly stained cells rising suspicion of biallelic *TP53* inactivation), MCT (to exclude SM-MDS)
AML	CD34 (to count blasts and detect dysplastic megakaryocytes) MPO (to prove usually dim and in ≥ 3%, and in APML with *PML::RARA* strong expression on blasts), mutational specific anti-NPM1 (defines AML, even if MPO is negative, and enables estimation the size of the mutated clone and—combined with histomorphologic analysis—counting blasts), CD15 and CD33 (to prove usually strong expression on blasts, if MPO and mutated NPM1 are negative), CD117 (to count blasts after subtraction of MCT + mast cells and E-cadherin + pro-erythroblasts, if CD34 and mutated NPM1 are negative), CD11c, CD14, CD68, CD117, CD163, Lysozyme, IRF8dim and/or mutated NPM1 (to count blasts with monopoietic differentiation), CD71, CD117, E-cadherin, glycophorin A, GLUT1 and/or—contextual—p53 (to count blasts with erythropoietic differentiation; usually *TP53* mutated) CD42b, CD61, vWF and/or—contextual—p53 (to count blasts with megakaryopoietic differentiation; often *TP53* mutated) p53 (to detect strongly stained cells rising suspicion of biallelic *TP53* inactivation) MCT (to exclude SM-AML, especially in AML with *RUNX1::RUNX1T1*) CD3, CD19, PAX5, CD303, TDF4 (to exclude ALAL or BPDCN)
ALAL	CD3 (ε-chain antibody), CD10, CD11c, CD14, CD19, CD22, CD79a, Lysozyme, MPO, PAX5 as recommended by the WHO-5 Mutated NPM1 must be negative
M/LN-Eo and TKGF	CD34 (to count blasts and analyze bone marrow microvessels), CD42b or CD61 (to detect abnormal megakaryocytes), MCT and CD25 (to detect increased and abnormal mast cells), CD71 or E-cadherin (to detect erythroid microtumors in M/LN-Eo with *PCM1::JAK2*)
BPDCN	CD123, CD303, strong IRF8, SOX4, TCF4, TCL1 (expression of 3 is defining) CD4, CD56, TdT (if CD123 and one of the above is positive, expression of the here listed markers is considered defining) CD3, CD14, CD19, CD34, Lysozyme, MPO (must be negative per definition)

Abbreviations: *aCML* atypical chronic myeloid leukemia, *ALAL* acute leukemia of ambiguous lineage, *AML* acute myeloid leukemia, *BPDCN* blastic plasmacytoid dendritic cell neoplasm, *CALR* calreticulin, *CML* chronic myeloid leukemia, *CMML* chronic myelomonocytic leukemia, *CNL* chronic neutrophilic leukemia, *MCT* mast cell tryptase, *MDS* myelodysplastic syndrome, *MDS/MPN* myelodysplastic-myeloproliferative neoplasm, *M/LN-Eo and TKGF* myeloid/lymphoid neoplasms with eosinophilia and tyrosine kinase gene fusions, *mPDC* mature plasmacytoid dendritic cell, *MPN* myeloproliferative neoplasm, *MPO* myeloperoxidase, *SM* systemic mastocytosis, *SM-AML* systemic mastocytosis with associated acute myeloid leukemia, *SM-AMN* systemic mastocytosis with associated myeloid neoplasm, *SM-CMML* systemic mastocytosis with associated chronic myelomonocytic leukemia, *SM-MDS* systemic mastocytosis with associated myelodysplastic syndrome

## Optimal work-up of a TBMB

To retain the diagnostic-analytic integrity of biologic macromolecules, i.e., proteins (at least relevant epitopes), DNA (at least fragments suitable for sequencing or ISH), and RNA (at least fragments containing relevant gene fusions), which is essential to improve the diagnostic yield, TBMB must be properly fixed and decalcified. Despite several fixatives and decalcification procedures available that may have some advantages respecting morphology or turn-over times, the preferred protocol for the best preservation of the above diagnostically decisive macromolecules in TBMB is fixation in 10% buffered formalin, i.e., 3.7–4% formaldehyde, pH 7.4, for 8–72 h, and decalcification with 10–14% ethylenediamine-tetraacetate (EDTA) for 8 (supported by machines) to 72 h [9, 44].

Standard histochemical stainings to be applied to TBMB, besides the standard hematoxylin and eosin (H&E) stain, include Gömöri (to assess argyrophilic myelofibrosis, MF), Giemsa (to assess erythropoiesis, cells with metachromatic granules, and plasma cells), periodic-acid-Schiff (PAS) (to better assess megakaryo- and myelopoiesis), and in selected cases, Masson’s trichrome (to assess collagen deposition and osteosclerosis) and Turnbull staining (to detect ferrous iron, which is more relevant than the ferric iron detected by Perl’s staining, yet with the caveats of iron depletion after any decalcification).

Along with IHC and ISH, genetic sequencing testing, including HTS, has become the diagnostic standard in many MN entities and can be achieved by dedicated “myeloid panels” that are applicable to—as described above—properly processed TBMB [13]. These technologies (IHC, ISH, sequencing) are essential pillars of a TBMB-based diagnostic approach.

## Diagnostic issues in the various entities of myeloid and precursor neoplasms

Taking into account (1) the clinical context including information on the spleen size, duration of symptoms, and exposure to myelotoxic or hematopoiesis-stimulating drugs, (2) the CBC, and (3) the histomorphology, a suspected diagnosis of a large array of MN and precursor neoplasms can be made. These include myeloproliferative- (MPN), myelodysplastic- (MDS), or myelodysplastic-myeloproliferative neoplasm (MDS/MPN), accompanied or not by systemic mastocytosis (SM), SM on itself, myeloid/lymphoid neoplasms with eosinophilia and tyrosine kinase gene fusions (M/LN-Eo-TKGF), precursor cell neoplasms, i.e., acute myeloid or lymphoblastic leukemia (AML or ALL), or blastic plasmacytoid dendritic cell neoplasm (BPDCN). To reach a specific diagnosis, a plethora of ancillary in situ and in vitro tests should be performed on the diagnostic TBMB as exemplified below.

### Myeloproliferative neoplasms

The diagnosis of MPN—especially in the setting of advanced myelofibrosis—mainly relies on the histopathological evaluation of the TBMB, as often the BM cells remain entrapped in the fiber network and cannot be aspirated. Indeed, evaluation of the TBMB is among the major criteria for the diagnosis of all MPN, including chronic neutrophilic and eosinophilic leukemia (CNL and CEL) [5]. Morphologically, many cases present with a hypercellular BM showing maturation of all three lineages. While in CML, the massive hyperplasia of the myelopoiesis, occasionally accompanied by an increase of dwarf megakaryocytes, is the clue to diagnosis, it might be more difficult to differentiate other forms of MPN from reactive changes or from one another. In essential thrombocythemia (ET), the cellularity of the BM should be within the age-adjusted range, and its morphologic hallmark is hypersegmented “stag-horn like” megakaryocytes being singly dispersed in the BM spaces. There should be no larger clusters of > 7 megakaryocytes. If these are seen and megakaryocytes show a more variable morphology, including a hyposegmented and hyperchromatic “cloudy” nucleus, the differential diagnosis of prefibrotic primary myelofibrosis (per-PMF) should be considered. In polycythemia vera (PV), there is markedly hypercellular BM (also referred to as panmyelosis) as well as hyperplasia of all three lineages. There might be small clusters of megakaryocytes; yet they should not show the atypia seen in PMF. In any MPN, fibrosis should be graded according to established criteria [28, 43].

TBMB might be obtained in known cases of MPN in which patients present with new onset of anemia, thrombocytopenia, monocytosis, or blast increase in the peripheral blood. In these cases, progression of MF and also MDS- or MDS/MPN-like evolution as well as transformation should be ruled out [18].

**Fig. 1:**
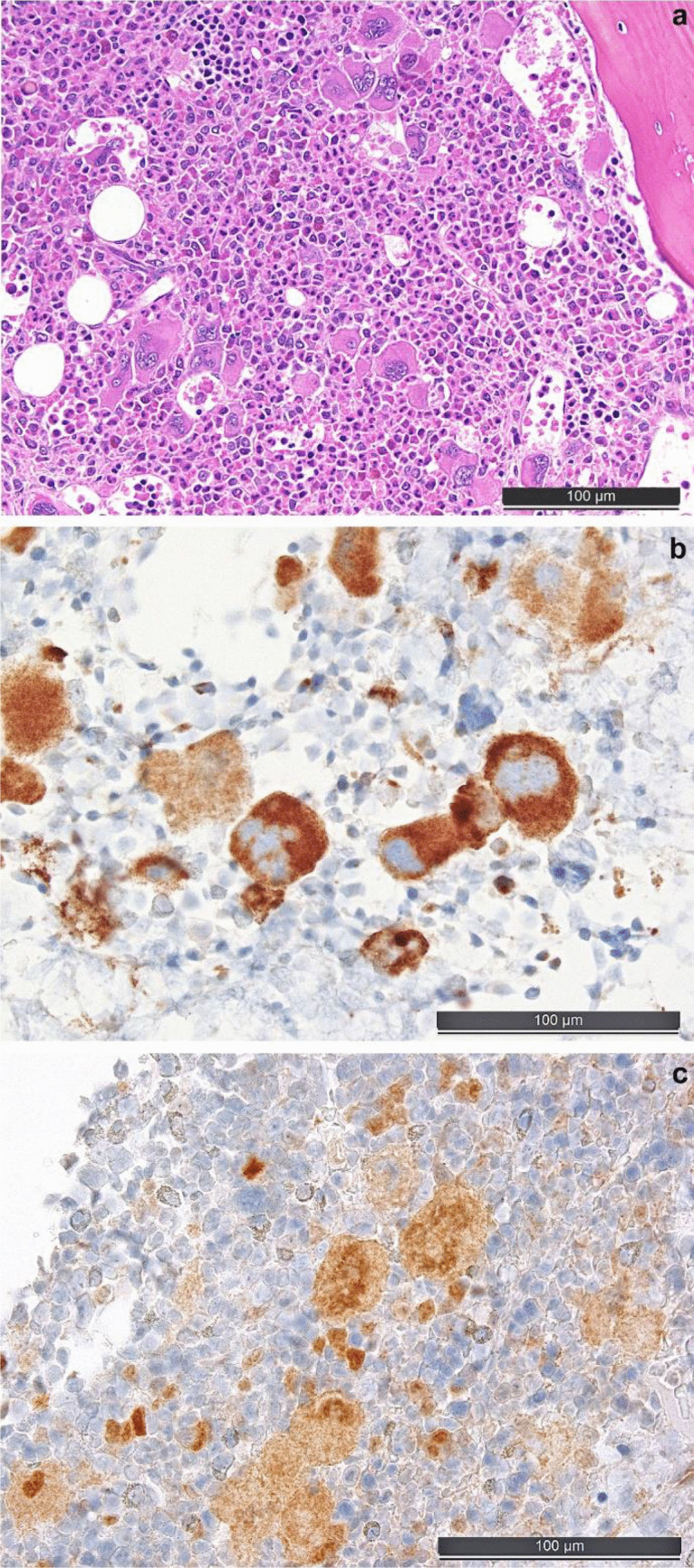
Bone marrow biopsy morphology and useful ancillary stainings in myeloproliferative neoplasms. **a** Prefibrotic primary myelofibrosis (PMF) that turned out to be calreticulin (*CALR*) mutated on molecular testing. **b** Staining with the mutational-specific anti-calreticulin antibody CAL2 in the case of *CALR*-mutated PMF. **c** Pathologic nuclear phospho-STAT5 positivity with the antibody clone 8–5-2 that recognizes the Y694 phosphoepitope of STAT5a and the homologous phosphoepitope Y699 of STAT5b in a *JAK2 *V617F-mutated PV; while the nuclear expression in erythroid precursors is physiologic, nuclear presence of phospho-STAT5 in megakaryocytes reflects highly active JAK-STAT signaling such as in the presence of MPN-driver mutations [1]

In suspect MPN, ISH to exclude/detect *BCR::ABL1* fusion, combined with the application of the mutation-specific anti-calreticulin antibody CAL2 [42] and anti-phospho-STAT5 antibody indicative of *JAK2 * and/or *MPL * mutations (see Fig. 1), combined with sequencing of the respective genes [1, 19] and sequencing for *CSF3R* mutations (to rule out CNL), would allow establishing entity-specific diagnosis in most cases. CEL would be in need of additional testing to particularly exclude SM and M/LN-Eo-TKGF.

With respect to differential diagnosis, several drug-induced changes of the hematopoiesis might mimic MPN. These primarily include the application of thrombopoietin agonists and G-CSF and EPO, which induce hyperplasia of the megakaryocytes, the myeloid, and the erythroid lineage, respectively [8, 24]. MPN-like changes can also be induced paraneoplastically by tumors producing these factors such as hepatocellular carcinoma, renal cell carcinoma, mesothelioma, or soft tissue sarcomas [2].

### Myelodysplastic syndromes/neoplasms

**Fig. 2:**
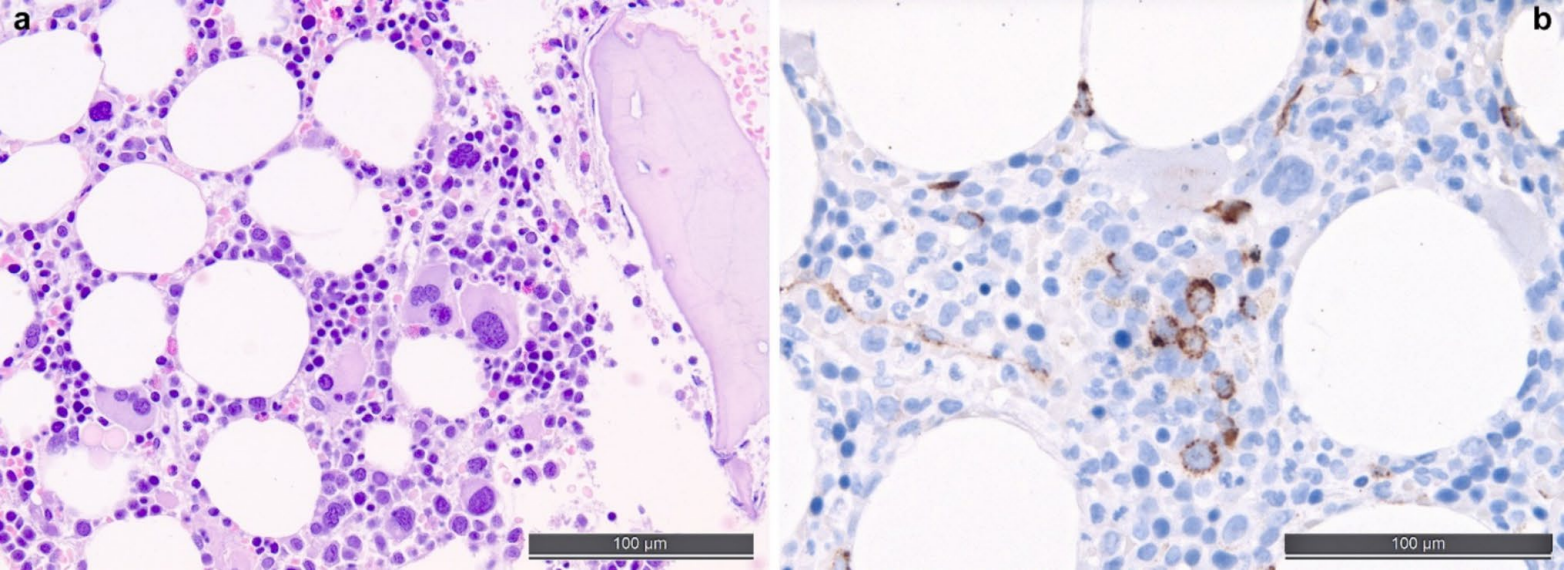
Bone marrow biopsy morphology and characteristic atypically located immature precursors in a myelodysplastic syndrome/neoplasm (MDS). **a** High-quality H&E-stained slide with unequivocal dysmegakaryopoiesis with peritrabecular displaced small megakaryocytes with hyperchromatic and separated nuclei, myeloid maturation disturbances and dyserythropoiesis with nuclear blebs and megaloblastic changes and peritrabecular displacement. **b** Atypically located immature precursors (ALIP), i.e., groups of ≥ 3 CD34 + cells in non-paratrabecular location, which are highly characteristic of MDS

MDS is the most important differential diagnosis in cases of unexplained cytopenias (except for MDS with del(5q) that may be accompanied by thrombocytosis). Yet, a diagnosis solely based on morphology is difficult, which has been recognized by both recent classifications of MN, partially omitting “dysplasia” as an indispensable prerequisite for the diagnosis of MDS [5, 27]. Nevertheless, on TBMB, atypia of megakaryocytes and dyserythropoiesis can be assessed by experienced hematopathologists (see Fig. 2a). Atypical megakaryocytes may show hypolobulated and hyperchromatic nuclei; micromegakaryocytes (size similar to promyelocytes) are also a strong indicator of dysplastic changes. Dyserythropoiesis is reflected by erythroid precursors that show irregular nuclear contours or nuclear protrusions. TBMB allows for the assessment of the deranged spatial distribution of the hematopoietic lineages too, e.g., peritrabecular megakaryocytes and displacement of myelopoiesis from the peritrabecular to the central subcompartment. Yet, it should be considered that almost all these changes could also be induced by toxic or drug exposure or vitamin deficiency. Of note, in cases of MDS with fibrosis, recognized as a specific subentity according to the WHO-5 [27], the complete diagnosis might again rely on the TBMB due to a dry tap.

For the further work-up of suspected MDS, an evaluation of the presence of blasts by IHC for CD34 is mandatory as this can group cases into categories with an increase of blasts (> 5–10% and > 10–20%, respectively), which has a huge implication on both the prognosis as well as the treatment options of patients. Counting the percentage of CD34-positive cells (consistent with blasts) out of 500 to 1000 cells (roughly corresponding to the visible cells in 1 hpf at 400 magnification, i.e., roughly 0.2 mm² of BMB with 30–70% cellularity) and reporting the presence and counts in the hot spots is advisable [44]. CD34 also helps to detect atypical localization of immature precursors (ALIP) foci, another feature of dysplasia. ALIP foci are defined by at least three CD34-positive myeloid cells forming an aggregate and lying in the intertrabecular space not close to the bony trabeculae [10] (see Fig. 2b). CD34 expression on megakaryocytes is also a feature of dysplasia. Detecting an increase of p53 expression might be indicative of *TP53* mutations. In our routine praxis, we refer to a threshold of strong expression of p53 of 2% of BM cells as suggested in the literature [34, 41], bearing in mind that in cases of *TP53 *nonsense and some frameshift mutations, which result in a complete loss of p53 protein expression, IHC may be false-negative, explaining the almost 100% specificity, but only 85% sensitivity of this technology to predict mutations [37].

A rare subtype of MDS is hypoplastic MDS, often mimicking aplastic anemia [32, 36]. Atypia of megakaryocytes and dyserythropoiesis might point at this differential diagnosis, and evaluating the TBMB by CD34 is absolutely essential to detect ALIP, aberrant expression of CD34 in megakaryocytes as well as an increase of blasts, as mentioned above.

Detecting typical MDS-associated mutations, which can reliably be done on DNA extracted from FFPE TBMB, in genes characteristically mutated in MN might strengthen the suspicion of MDS. However, the differential diagnoses of CHIP and CCUS when the CBC is nearly normal or in the absence of clear-cut MDS criteria must be kept in mind.

Since two MDS subentities are defined by either *SF3B1* or *TP53* mutations, respective testing at least for mutations of these two genes is mandatory [5, 27]. In cases with *SF3B1* mutations, the presentation with concomitant ring sideroblasts might be a useful hint.

Sometimes, reactive changes are very difficult to almost impossible to distinguish from MDS-related changes if only the TBMB is available. In such cases, especially those with low blast counts, a comment should be added referring to the essential correlation with the patient’s drug-exposure history (e.g., administration of methotrexate, azathioprine, chemotherapeutic drugs) as well as with the findings of BM cytology (only here dysplasia of the myeloid cells can be sufficiently appreciated), flow cytometry (Ogata score), and genetic analysis (mutations as well as structural chromosomal aberrations).

### Myelodysplastic-myeloproliferative neoplasms

**Fig. 3:**
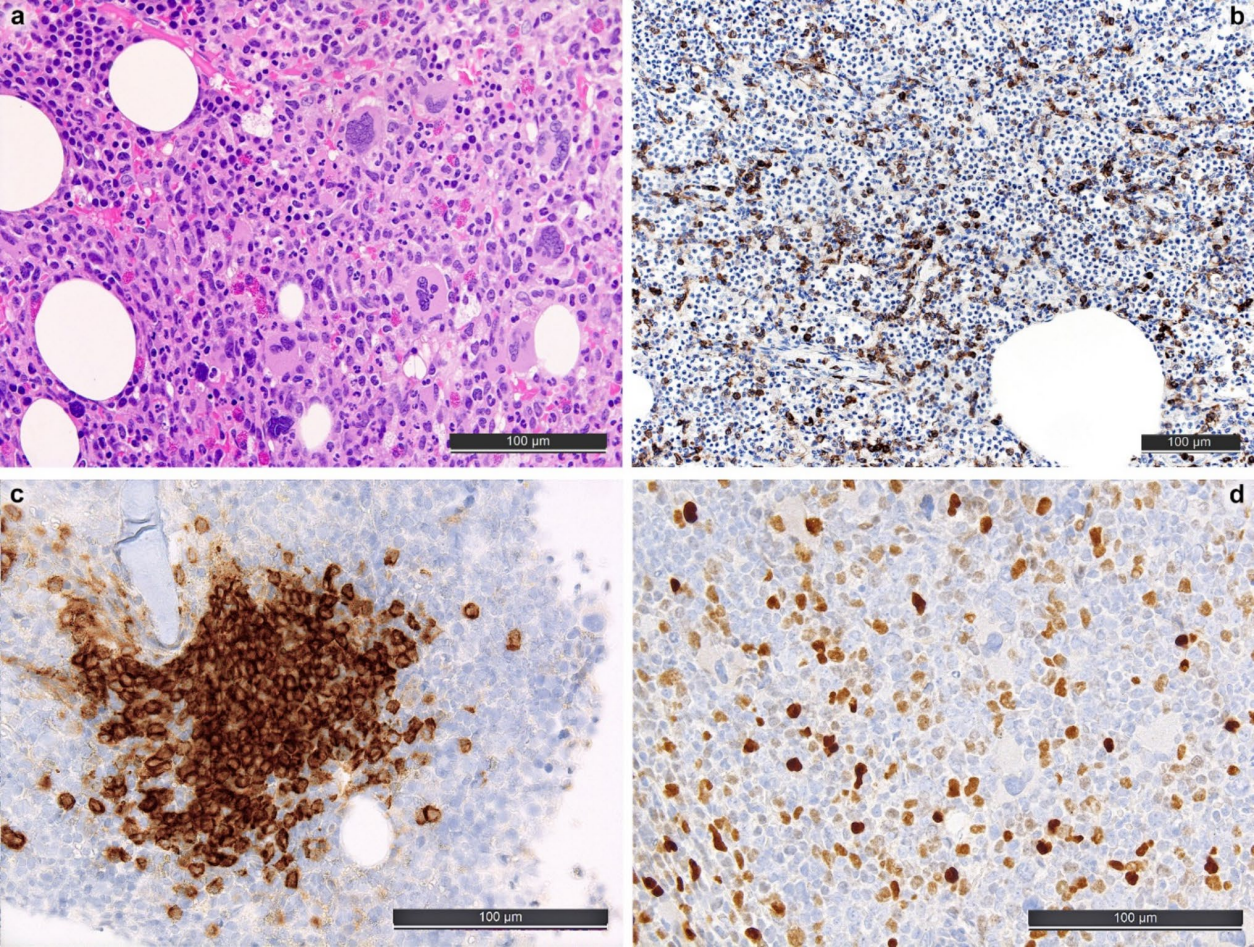
Bone marrow morphology and characteristic immunohistochemical findings in chronic myelomonocytic leukemia (CMML). **a** High-quality H&E-stained slide with hyperplastic, myeloid-predominant, maturating hematopoiesis with megakaryocytic atypia (to a part MPN-like with cloudy staghorn-like nuclei, to a part MDS-like with small form with hyperchromatic lobate nuclei). **b** CD14-staining showing ≥ 10% increase of monocytes, characteristic of CMML [40]. **c** CD123-staining showing mature plasmacytoid dendritic cell (mPDC) nodule, characteristic of CMML [35]. **d** IRF8-staining showing strongly stained mPDC and dim-stained monoblast equivalents in CMML [26, 45]

In MDS/MPN overlaps, both features of myeloproliferative (cytosis, increased hyperplastic hematopoietic cells) as well as myelodysplastic (cytopenia, atypia of megakaryocytes and erythroid precursors, myeloid maturation disturbances) features are seen at varying degrees. Correlation with the CBC is vital not to overlook cases of chronic myelomonocytic leukemia (CMML); another clue to this entity is mature plasmacytoid dendritic cells forming nodular aggregates that can be detected by IHC for CD123 and/or IRF8 [45] (see Fig. 3). An increase of monocytes in the BM can be verified by staining for CD11c, CD14, or CD68, with CD14 showing the best results [35, 40, 46]. By careful assessment of the IRF8-stained slides for cells displaying weak nuclear positivity, one may roughly estimate monoblast equivalents [26]. Importantly, TBMB findings have been recognized as a criterion for the diagnosis of CMML [5]. In all cases of CMML (and MDS/MPN, NOS), SM (see also below) should also be ruled out as it can present as very subtle infiltrates difficult to detect by morphology alone.

At the genetic level, *TET2* and *SRSF2* (*CBL, SETBP1*) or multiple *TET2* co-mutations strongly indicate CMML, while concurrent *ASXL1* and *SETBP1* mutations in the absence of MPN-driver variants, absence of monocyte increase, and strongly myeloid-predominant hematopoiesis are suggestive of aCML/MDS/MPN with neutrophilia. Finally, MPN-driver mutated cases (especially with high variant allelic frequency) with *RAS*-family co-mutations are suspicious of MPN with MPN/MDS-like progression (see below), and a history of MPN or MDS tends to exclude some MDS/MPN subtypes such as MDS/MPN-T-*SF3B1* and NOS [15, 18].

In other types of MPN/MDS, a diagnosis solely based on the evaluation of the TBMB is impossible as either specific genetic alterations are essential for the diagnosis (such as *SF3B1* mutations) or criteria that cannot be assessed on the TBMB must be fulfilled, such as a specific type of maturation disturbances or dysplasia.

Besides cases classified as spectrum MPN/MDS overlaps, progressions of MDS with MPN-like features and progressions of MPN with newly appearing dysplastic or MDS/MPN features should be considered [18]. Especially in the latter category, typical stigmata of MPN (PMF-like megakaryocytes with advanced myelofibrosis and osteosclerosis) can easily be detected in the TBMB. IHC stains for CD34 (to rule out blast increase and to detect enlarged sinuses with intrasinusoidal hematopoiesis), CD61 (to detect micromegakaryocytes and clusters), p53 (reflecting *TP53* alterations as indicators of disease progression), mutated calreticulin, and phospho-STAT5 (to detect nuclear expression in the [majority of] megakaryocytes as a readout of MPN-driver mutations) are helpful addenda in the work-up of these biopsies.

Reactive monocytosis is an important differential diagnosis of CMML [30]. Other relevant differential diagnoses include hemophagocytic lymphohistiocytosis (HLH), granulomatous and infectious diseases (leishmaniosis, histoplasmosis, etc.) which may all be accompanied by monocyte increase and should be accounted for.

### Acute myeloid leukemia

In many cases, the morphologic diagnosis of AML is rather straightforward as the diffuse increase of blasts, in many cases excessively exceeding the amount of normal hematopoiesis, can be readily seen. The hematopoiesis in the background should nevertheless be examined carefully as dysplastic changes might allude to the differential diagnosis of an AML in the background of MDS, which may be indicative of a special clinical course [6], and there might be accompanying other disorders such as SM (especially in AML with *RUNX1::RUNX1T1* fusion) or plasma cell neoplasm.

It is vital to characterize the blasts by immunohistochemistry. Many AML cases show expression of CD34 and are also positive for myeloid markers such as CD117 and myeloperoxidase. However, those with monopoietic differentiation are negative for CD34. Here, monoblastic markers such as IRF8 might be helpful, along with other (largely) monocytic markers (CD11c, CD14, CD56, CD68).

**Fig. 4:**
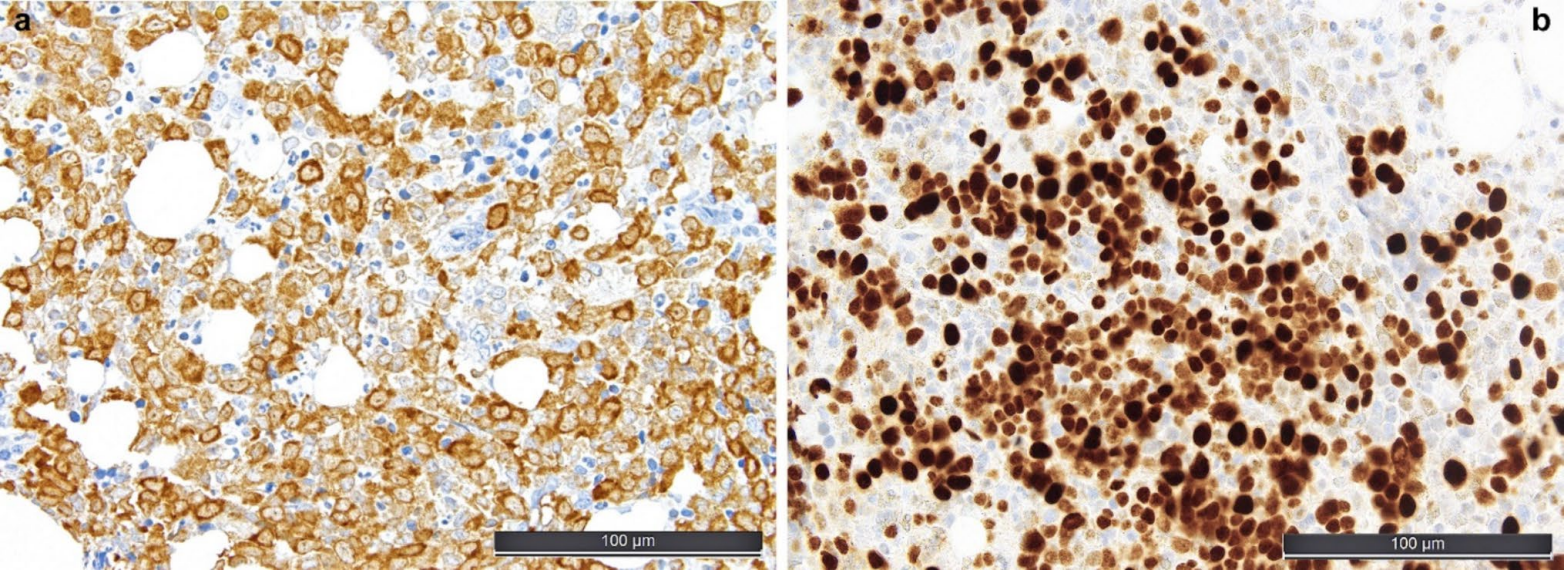
Useful ancillary stainings in acute myeloid leukemia (AML). **a** Diffuse cytoplasmic positivity with the mutational-specific anti-NPM1 antibody PA1-46356 in a respectively mutated AML. **b** Strong staining for p53 in approximately 50% of cells in a *TP53* mutated AML; for explanatory notes regarding interpretation of the p53 staining, the reader is referred to the further ahead paragraph on myelodysplastic syndromes/neoplasms

However, immunohistochemical characterization might also include some pitfalls: in the common subtype of AML with *NPM1 * mutations (which also often shows some degree of monopoietic differentiation), CD34 is consistently negative; here, the use of a mutation-specific NPM1 antibody has proven to be very useful, and it can also be used as a sensitive method to detect minimal residual disease [39] (see Fig. 4a). The application of a mutation-specific antibody is preferred since, yet missing rare mutational variants in exon 5, it recognizes the result of the 4-base pair insertion at position 863/864 in types A, B, and D mutations and does not recognize the ubiquitously present wild-type protein, enabling an easy readout without requiring specific subcellular (cytoplasmic) detection of the potentially mutated protein that has otherwise to be discerned from the wild-type nuclear one, which can be very difficult to impossible in blasts with scant cytoplasms.

In AML with *RUNX1 *alterations, the blasts can show expression of CD19, CD79a, and/or PAX5 [31] which might lead to the suspect diagnosis of B-ALL or MPAL. In this case, further myeloid (myeloperoxidase, CD33, CD117) and lymphoid markers (CD20, CD22), as well as correlation with flow cytometry results and mutational analysis, should be sought to resolve this dilemma. Along these lines, weak expression of myeloperoxidase can be seen in a few cases of B-ALL [4]. AML blasts can also express CD123, which needs to be differentiated from an increase of plasmacytoid dendritic cells or blastic plasmacytoid dendritic cell neoplasms (BPDCN). Expression of mutated NPM1 by IHC in the context of negativity for other BPDCN markers (see below), which is often seen in AML with CD123 expression, is a helpful tool in this differential diagnosis. In any case of AML, the results obtained on the aspirate smears should be compared with the number of blast equivalents obtained on TBMB. Especially in cases that are technically not assessable, e.g., cytolytic or hemodiluted aspirates, dry taps, BM diseases with substantial fibrosis, and in CD56 + MN blast-equivalent numbers must be enumerated on the TBMB [12].

Diagnosis of several types of AML defined by *CEBPA*-, *NPM1*-, *TP53*-, or MDS-related mutations is based on sequencing results (see Fig. 4b), while other mutations such as (e.g., *IDH1* or *IDH2*) are linked to drug sensitivities, and therefore HTS must be applied in respective instances, which is feasible for FFPE material, which may be the only source of DNA in specific settings as those mentioned in the paragraph above [11]. Efficiently, the mutational-specific anti-NPM1 antibody PA1-46356 enables identification of this most common AML type [38].

### Systemic mastocytosis

SM morphologically often presents as subtle paratrabecular or, more rarely, interstitial infiltrates of epithelioid or spindle cells. Identification of > 14 mast cells in aggregates detected in TBMB and/or biopsies from other extracutaneous organs is the major diagnostic criterion for SM. In the Giemsa stain, mast cells can be identified by their granulation; yet, mast cells can also be degranulated and thus more difficult to identify. Therefore, it is highly recommended to apply ancillary IHC for tryptase and CD117. In any MN, but especially in CMML, MDS/MPN, NOS, and AML *RUNX1::RUNX1T1*, careful examination of the TBMB to exclude accompanying SM is mandatory.

**Fig. 5:**
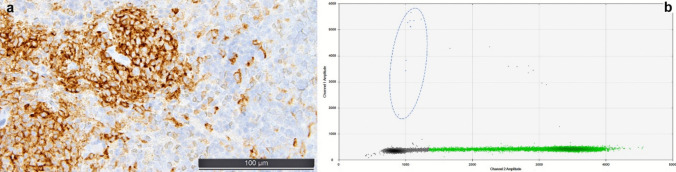
Ancillary diagnostic techniques in systemic mastocytosis (SM). **a** CD30-staining of mast cell infiltrates of an aggressive SM. **b** Digital droplet PCR visualization of the presence of 0.023% *KIT* D816F-mutated alleles (few spots within the area of the dotted line in the left part of the diagram), compared to 99.977% wild-type alleles (myriad of green dots) out of a sample of DNA extracted form a BM biopsy of a patient 24 months after allogeneic hematopoietic transplantation for SM-AMN and fractioned into 20,000 droplets; non-informative droplets are delineated in grey; these alleles were not detected by the same technology applied to the aspirate

Aberrant expression of CD2, CD25, and CD30 and mutations of *KIT* are features of minor criteria for the diagnosis of SM, which can be accrued by analysis of the TBMB (see Fig. 5a). Especially in cases with only subtle SM-infiltrates, *KIT* sequencing analysis using sensitive techniques such as digital droplet PCRs can detect activating *KIT* mutations at a variant allelic frequency of < 0.1% (see Fig. 5b).

### Myeloid/lymphoid neoplasms with eosinophilia and tyrosine kinase gene fusions

**Fig. 6:**
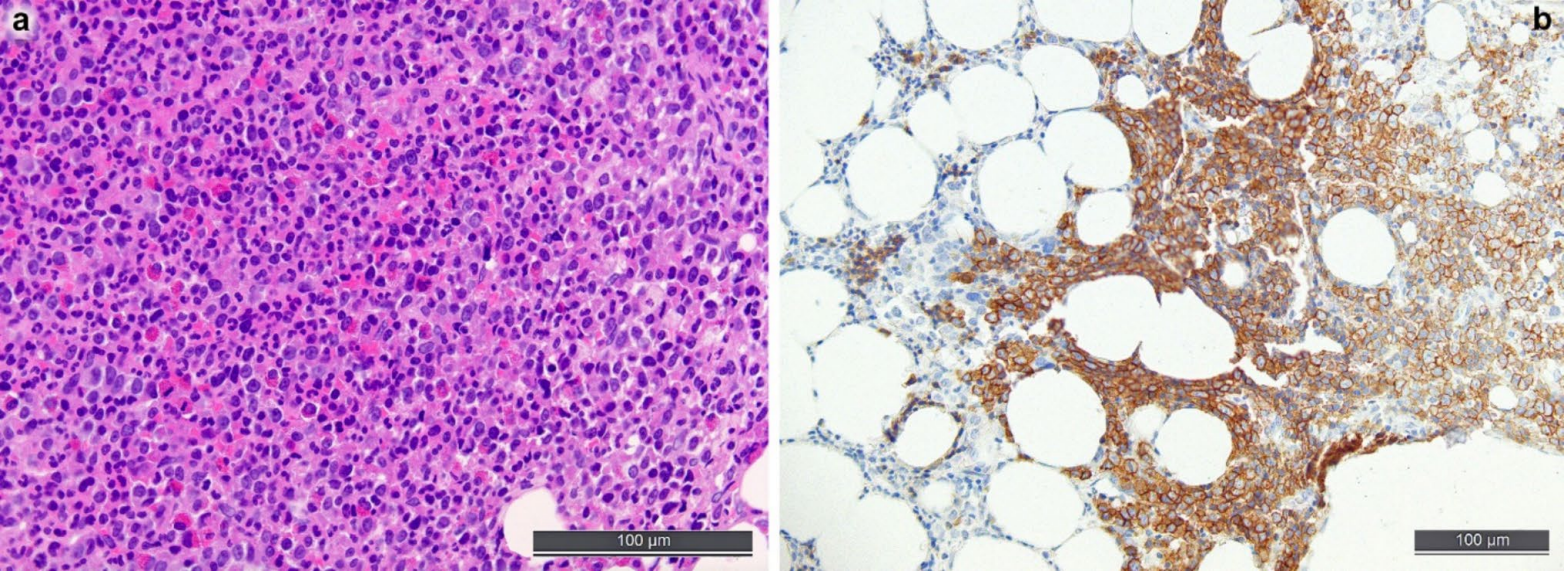
Characteristic bone marrow biopsy appearance myeloid/lymphoid neoplasms with eosinophilia and tyrosine kinase gene fusions. **a** High-quality H&E-stained slide with hyperplastic, myeloid-predominant, maturating hematopoiesis with perceptible eosinophilia. **b** Megaloblastic erythroid microtumor (compared with a small cell erythron in the left-hand-middle/9h part of the image) highly predictive of *PCM1::JAK2* fusion [22], highlighted by E-cadherin-staining

Particularly in male patients presenting with MN with eosinophilia, monocytosis, MF, mast cell (even CD25 +), and/or lymphoblast excess, accompanying extramedullary myeloid proliferations (lymph nodes and/or myeloid sarcomas), M/LN-Eo-TKGF should be suspected. Unfortunately, the morphology of MLNEs is rather non-specific (see Fig. 6a) and correlation with clinical data and CBC is essential. In the TBMB, most cases present striking eosinophilia, myeloid-predominant hematopoiesis, subtle dysplastic changes of the megakaryopoiesis, and MF. One exception is the presence of “erythroid microtumors” (especially in the context of eosinophilia, MF, male gender, MPN, or MDS/MPN-like presentation), which is virtually pathognomonic and should prompt a presumptive diagnosis of M/LN-Eo with *PCM1::JAK2*-fusion [22] (see Fig. 6b). Further immunohistochemical analyses can be performed to better assess hematopoietic lineages, dysplastic changes, and an excess of blasts (both myeloblasts and lymphoblasts). To establish the specific diagnoses of the various subtypes of M/LN-Eo, sensitive molecular RNA-based testing to detect respective tyrosine kinase gene fusions must be applied, as based solely on morphology, the differential diagnoses remain widespread, including MPN, MDS/MPN, ALL, SM, and CEL [47].

### Blastic plasmacytoid dendritic cell neoplasm

BPDCN involves—beyond the skin—the BM in the majority of cases already at the time of initial presentation. The neoplastic cells can have a variable appearance ranging from monocytoid cells with irregular nuclei to small blastoid and larger blastoid cells resembling immunoblasts (especially in *MYC*-rearranged cases) [22].

**Fig. 7:**
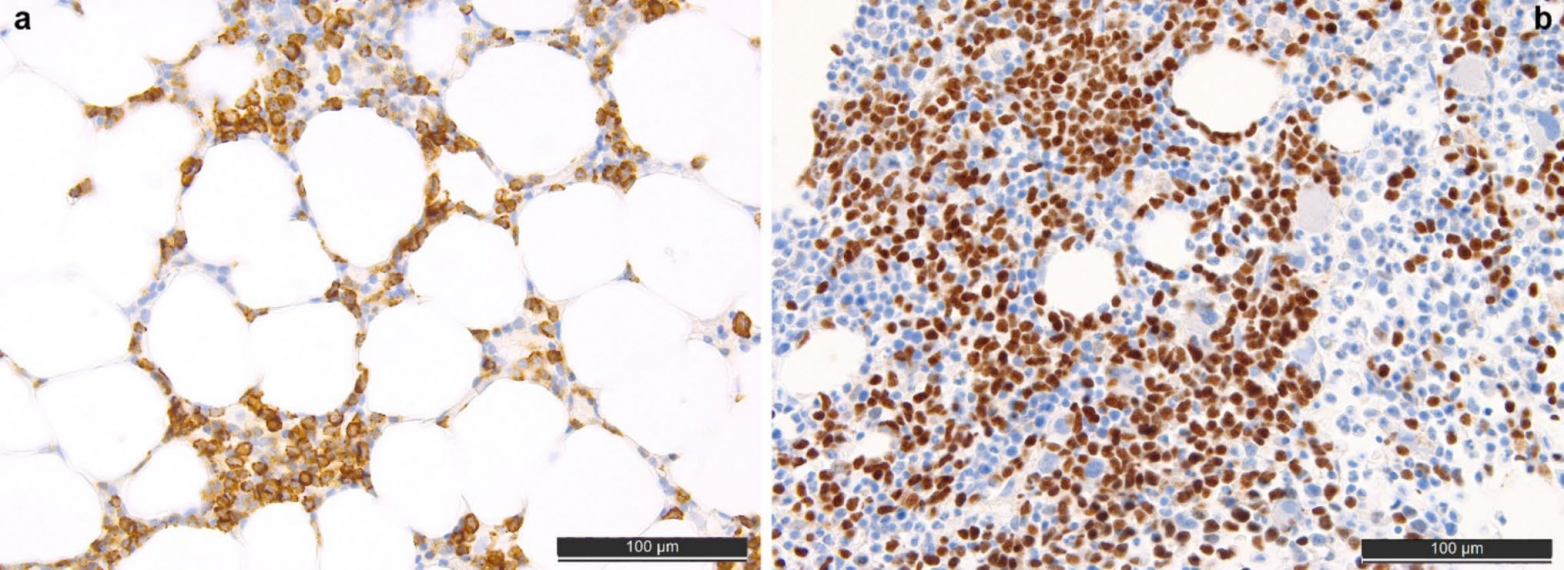
Useful ancillary stainings in blastic plasmacytoid dendritic cell neoplasms (BPDCN). **a** CD123-staining showing interstitial infiltrates of small blastoid cells, in the context of their additional positivity for CD303 and TCL1 and negativity for CD3, CD14, CD19, CD34, lysozyme, MPO (not shown), diagnostic of BPDCN. **b** Typical strong IRF8-staining of BPDCN [45]

BPDCN should be excluded in suspected cases and in any CD123 + MN by application of IHC for CD3, CD20, CD34, lysozyme, and myeloperoxidase that must be negative, and CD4, CD56, CD68, CD123, CD303, CD304, IRF8, SOX4, TCL1, TCF4, and TdT that can (at least two of them should) be positive [14, 16] (see Fig. 7).

In case “blastic plasmacytoid dendritic cells” show expression of mutated NPM1 or are *NPM1* mutated (assessed by sequencing), they should be classified as blasts of AML with *NPM1* mutation [17, 20]. Furthermore, an increase of rather mature plasmacytoid dendritic cells can be seen in a variety of T-cell lymphomas, in MDS, AML, and classically in CMML, in the latter three being part of the clone [7, 29, 48].

### Acute lymphoblastic leukemia

Similar to AML, the diagnosis of ALL is straightforward in most cases due to the diffuse infiltrates of blastoid cells. In contrast to myeloid blasts, the chromatin is more condensed, and nucleoli are inconspicuous.

Again, phenotypic characterization of the blasts is essential to come to a correct diagnosis. Besides CD34, TdT is expressed in most cases of ALL. These markers are also helpful to rule out infiltrates of mature lymphomas that can mimic ALL, such as Burkitt lymphomas or high-grade B-cell lymphomas NOS, which lack expression, especially of CD34. Lineage-specific markers for B-cells or T-cells should be assessed in any suspect ALL: expression of either or combined CD2, CD3, CD5, CD7, TCRβF1, or δ for the T-cell lineage, and CD19, CD20, CD22, CD79a, or PAX5 for the B-cell lineage. To avoid potential diagnostic pitfalls, it is important to note that dim myeloperoxidase staining in B-ALL [4], weak CD79a expression, and CD117 in early T-precursor ALL (ETP-ALL) is allowed [21, 23, 33], CD2 and CD7 can be expressed on AML blasts, and that *RUNX1* mutated or translocated AML can express CD19, CD79a, and/or PAX5 (see above).

As a last step, a WHO-5/ICC 2022 diagnosis should be established, which will require phenotypic and genotypic studies (e.g., FISH or RNA sequencing for cases of ETP-ALL with *BCL11B* rearrangement, or B-ALL with *BCR::ABL1*- or *ETV6::RUNX1*-fusions, and *MLL-*,* NUTM1-*, and *MYC-*rearranged instances), but subentity-specific diagnosis may not be feasible/achievable with the diagnostic tools disposable for TBMB.

**Fig. 8:**
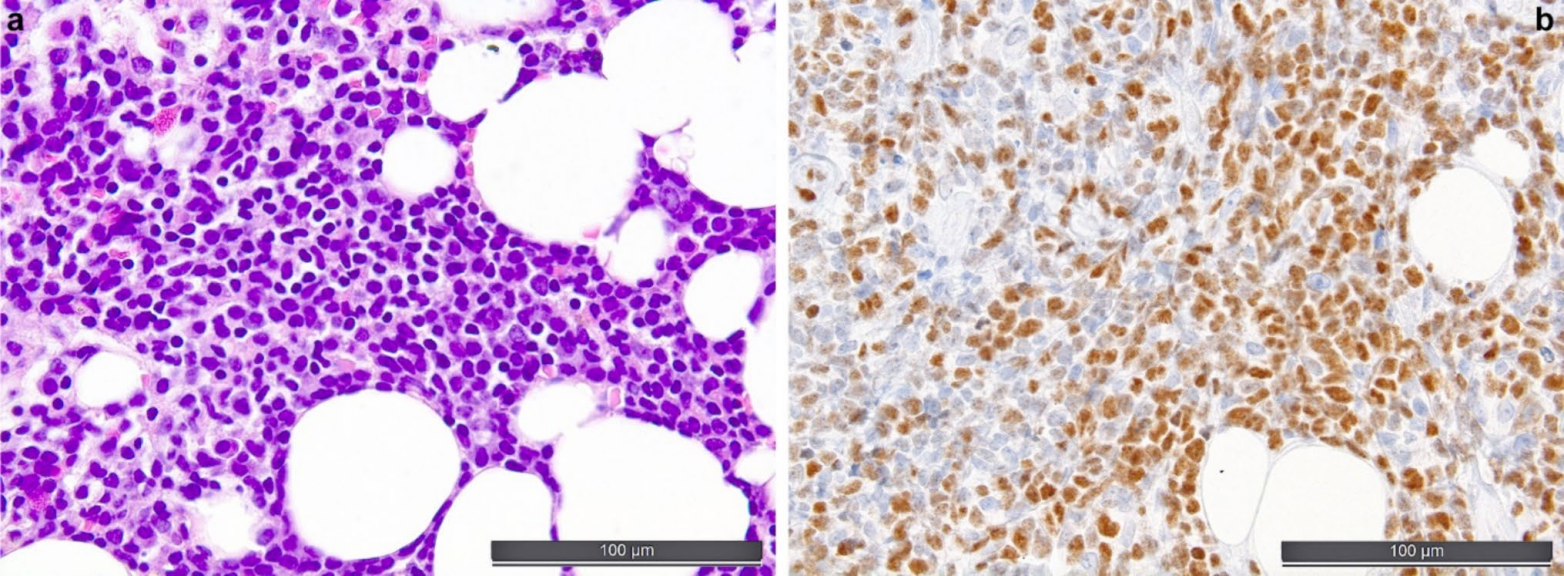
Acute lymphoblastic leukemia (ALL). **a** Interstitial and diffuse bone marrow infiltration by T-ALL. **b** Diagnostically useful overexpression of NOTCH1 in *NOTCH1*- mutated T-ALL [25]

When assessing an IHC-stain for TdT, a large number of hematogones, which are also positive for TdT and B-cell markers, might be misleading in suspecting B-ALL or, in the follow-up setting, of a B-ALL relapse. Hematogones are precursors of B-cells, which are mostly diffusely dispersed in the BM, not forming larger clusters. By morphology, they are very similar to lymphoblasts. Although the immunophenotype of lymphoblasts and hematogones is similar, the latter usually show a gradual/non-uniform staining for markers with a proportionately increased staining for CD34 (lowest amount of cells), TdT, CD20, and PAX5 (highest amount of cells), which has been described as a clue to distinguish hematogones from B-ALL blasts [3]. Furthermore, aberrant marker expression of B-ALL blasts, as well as genetic analysis, can assure the diagnosis of hematogones instead of B-ALL blasts, e.g., in the follow-up setting. In a small-blastoid B-cell background, expression of CD34, CD99, cytokeratin, ERG, dim myeloperoxidase, NUT1, and SOX11 is indicative of a pathologic immature phenotype and will help to discern neoplastic precursor cells from hematogones and mature lymphomas, while in the same context for T-cells, these are BCL11B, CD1a, CD10, CD34, CD117, LMO2, NOTCH1 (see Fig. 8), TTF1 (gene *NKX2.1*), and PU.1 (gene *SPI1*).

## Conclusions

TBMB is one of the essential requisites for achieving an adequate diagnosis of neoplastic hematologic diseases. In this review, we pointed out several specific requirements for the work-up of the TBMB as well as shared several diagnostic pearls and pitfalls focusing on MN and precursor neoplasms.

Although much information can be obtained from the TBMB alone, taking into account morphology and the ancillary techniques applicable to TBMB described above, it is vital to point out the need for an integrative character of the evaluation of the BM in which none of the different pillars is standing alone.

Such an approach, yet still not a reality in many countries, shows the biggest benefit for patient care by reaching correct diagnoses and thus opening the door for adequate treatment.

## Data Availability

No datasets were generated or analysed during the current study.
